# Evaluation of anatomical variants and pathological findings of the maxillary sinus prior to sinus floor elevation: A Cone Beam CT retrospective study in 660 patients

**DOI:** 10.4317/jced.61624

**Published:** 2024-06-01

**Authors:** Maria Barka, Catherine Donta, Spyros Damaskos, Emmanouil Chatzipetros, Christos Angelopoulos

**Affiliations:** 1 DDS, MSc. Resident, Department of Oral Diagnosis and Radiology, Faculty of Dentistry, National and Kapodistrian University of Athens, Athens, Greece; 2 DDS, PhD. Associate Professor, Department of Oral Diagnosis and Radiology, Faculty of Dentistry, National and Kapodistrian University of Athens, Athens, Greece; 3 DDS, PhD. Assistant Professor, Department of Oral Diagnosis and Radiology, Faculty of Dentistry, National and Kapodistrian University of Athens, Athens, Greece; 4 DDS, MSc, PhD. Research Associate, Department of Oral Diagnosis and Radiology, Faculty of Dentistry, National and Kapodistrian University of Athens, Athens, Greece; 5 DDS, MSc, PhD. Professor, Department of Oral Diagnosis and Radiology, Faculty of Dentistry, National and Kapodistrian University of Athens, Athens, Greece

## Abstract

**Background:**

Maxillary sinuses may present a wide spectrum of anatomical variations and pathological lesions whose recognition is mandatory for the clinician, especially prior to any surgical intervention in the area, such as in cases of sinus floor elevation. The goal of this study was the evaluation, mapping and prevalence of sinus anatomical variants and pathoses in Cone Beam Computed Tomography (CBCT) scans of dental patients.

**Material and Methods:**

660 CBCT examinations of adult patients involving both sinuses were obtained (between 2017 and 2023) and analyzed. The following anatomic variants and pathologic findings were evaluated: antral septa (AS), posterior superior alveolar artery (PSAA), sinus hypoplasia, sinusitis, odontogenic cysts, retention cysts, antroliths, and various less common lesions (e.g.: benign fibro-osseous dysplastic lesions, neoplasms etc.). Investigation of the association of the findings with patients’ age and gender was performed using the Chi-square test (X2), Fisher’s exact test, t-test, and ANOVA (P<0.05). Differences in prevalence between findings, based on their location (right and/or left sinus), were investigated using z-test and t-test.

**Results:**

AS were found in 38.6%, and PSAA was identifiable in 90.2% (mean distance from the sinus floor=6.44 mm) of the patients respectively. 3% of patients had hypoplasia, 15.6% sinusitis, 2.7% odontogenic cysts, 16.1% retention cysts, 8.6% antroliths, and 1.7% uncommon lesions (e.g. malignant neoplasms and fibrous dysplasia). A statistically significant association was found between gender and PSAA diameter, PSAA distance from the sinus floor, hypoplasia, sinusitis, and retention cysts, as well as a statistically significant association between age and PSSA diameter and sinusitis.

**Conclusions:**

The prevalence of various anatomical variants and pathologic findings of the maxillary sinus did not differ based on their location. However, some of these findings appear to be related to either gender or age. CBCT is a valuable diagnostic tool to identify various anatomical variants and pathological findings of the maxillary sinus.

** Key words:**Sinus, anatomy, pathology, CBCT.

## Introduction

The maxillary sinuses are an area of particular interest that presents a plethora of anatomical variants and pathological conditions. Their presence may affect surgical planning such as surgically elevating the sinus floor by increasing the height of the maxillary alveolus, when required, for implant placement (1). Notably, this has now become a very common and predicTable surgical procedure, regardless to the fact that the most frequent intraoperative complication during this elevation procedure is perforation of the Schneiderian membrane, which occurs in 11% to 56% of cases (1).

However, the presence of bony septa within the sinus, their orientation and height may increase the risk of this particular complication (2-5). Another important anatomical limitation is the presence of the posterior superior alveolar artery (PSAA - that supplies the lateral sinus wall and overlying membrane), and its injury during the creation of the bony window may, in some cases, lead to intraoperative bleeding. This results in a compromised field of vision for the surgeon and an increased risk of perforation of the Schneiderian membrane, as well as prolonging operative time (6,7).

In addition, the presence of pathology in maxillary sinuses, depending on its severity and extent, may be a contraindication for sinus floor elevation. Therefore, detailed preoperative imaging of the region is a prerequisite for successful surgical intervention. Cone-beam Computed Tomography (CBCT) is an imaging tool with a significant contribution to the preoperative assessment of maxillary sinuses, providing high-resolution imaging of the bony structures with low radiation doses for the patient (8).

This retrospective study aims to contribute valuable information to the evaluation of anatomical variants and pathology of the maxillary sinus in terms of type, location, dimensions, and distribution. It also focuses on the prevalence of these variants and their potential associations with age and gender, using CBCT scans of dental patients of various diagnostic protocols, prior to sinus floor elevation.

## Material and Methods

-Study sample

In our study, 4136 consecutive CBCT examinations - from January 2017 to February 2023 - were retrospectively analyzed. These were from the archive of the Department of Oral Diagnosis and Radiology, School of Dentistry, National and Kapodistrian University of Athens (NKUA), Greece. All CBCT examinations of patients; a) aged >18 years and b) both maxillary sinuses were imaged in their entirety were included in this study. Patients with a) genetic syndromes (e.g., cleft palate, facial asymmetry, or diseases affecting the size of the sinuses), b) a history of trauma or previous surgical interventions in the region of interest, and c) those of poor diagnostic information (e.g., artifacts affecting the area of interest, excessive motion artifacts, limited Field of View etc.) were excluded from this study.

After power analysis (significance criterion: α=0.05, power≥0.80%), a total of 660 consecutive CBCT examinations (1320 sinuses) that met the inclusion and exclusion criteria were finally evaluated.

This research protocol was approved by the Ethics Committee of the School of Dentistry under protocol number 597/15-06-2023, ensuring that all procedures were conducted in accordance with the principles of the Ethics Regulation of the Research Committee of NKUA. All participants have provided written consent to use their personal data for research purposes.

-CBCT image acquisition and analysis

All CBCT examinations were performed using Newtom VGi Dental Volumetric Tomograph (QR, Cefla, Verona, Italy - serial No VG17004S) at the Department of Oral Diagnosis and Radiology, School of Dentistry, NKUA. The focal spot was 0.3mm and kVp was fixed and preset at 110 kV. The mA used was variable as it was automatically determined by the machine using SafeBeam technology which allows optimal use of mA based on the density of the irradiated volume. Exposure time was 3.6 seconds, and voxel size was 0.3mm for standard resolution scans, while the corresponding values for high-resolution (HiRes) scans were 5.4 seconds and 0.15mm, respectively. The used field of view (FOV) was: 8x8cm, 12x8cm, 15x12cm, or 15x15cm, depending on the indication for each examination. Images were studied on a FlexScan MX210 color LCD monitor in low-light conditions using NNTsoftware®(version 7.2 - installation package: 7.2.0). Observations were made by consensus of a panel of three observers specialized in Oral and Maxillofacial Radiology.

The following anatomical variants and pathologic findings were evaluated: 1) antral septa (AS), 2) PSAA localization, 3) sinus hypoplasia, 4) sinusitis (odontogenic and non-odontogenic), 5) odontogenic cysts, 6) retention cysts, 7) antroliths, 8) less common lesions (e.g., benign fibro-osseous lesions, neoplasms, etc.).

Criteria used in the assessment of AS and PSAA that influence any potential sinus floor elevation surgical planning.

**Figure 1:**
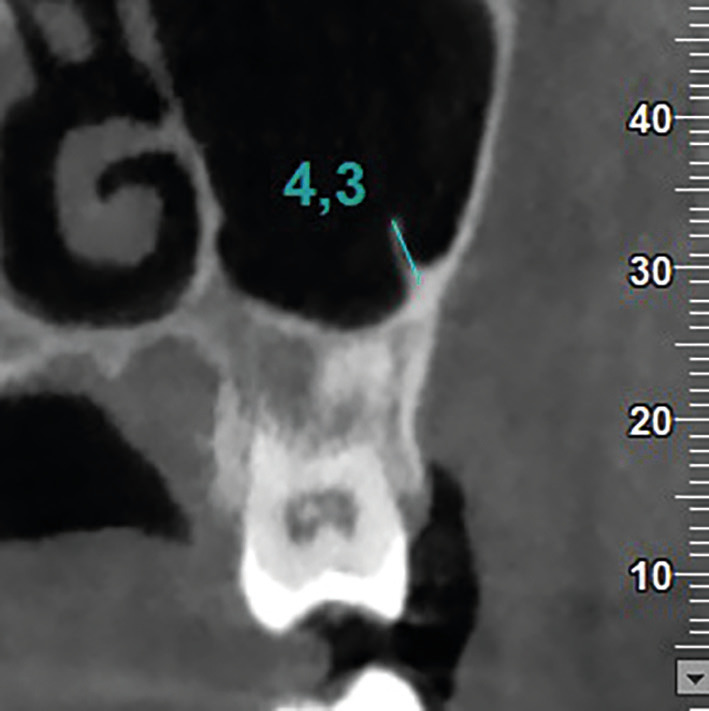
Coronal CBCT image illustrating the methodology for measuring AS height in a case of an AS originating from the inferior half of the lateral wall of the sinus.

**Figure 2:**
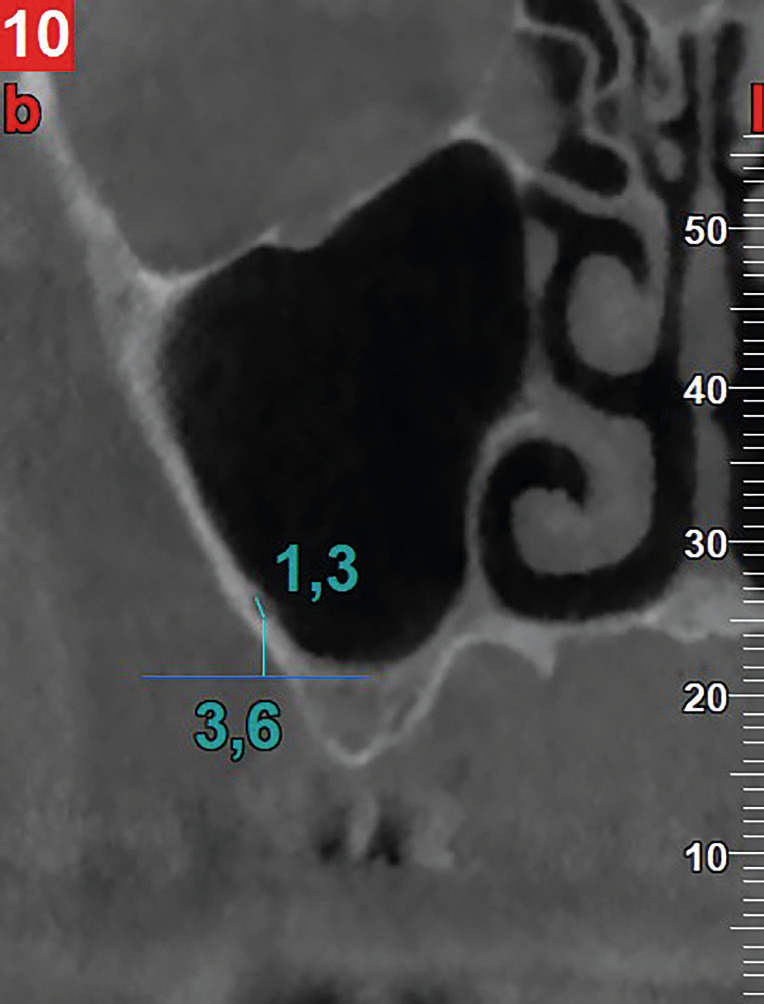
Coronal CBCT image illustrating the methodology for measuring the diameter of the PSAA and its distance from the sinus floor.

• AS: AS were observed in axial, coronal and sagittal sections and assessed for potential sinus floor elevation surgical planning. A minimum height of 2.5mm was set as a threshold (9). AS originating from the floor or the inferior half of the lateral walls of the sinus in the area between the first premolar and the second molar (i.e., in an area that could be involved in possible surgical elevation of the sinus floor) were evaluated (Fig. 1).

• PSAA: Observation was performed in coronal sections with a fixed magnification of 250%. The parameters evaluated included the visibility or not of the canal. In cases where the canal was visible, its diameter (<1mm or ≥1mm)10 was measured, along with the distance from its lower border to the sinus floor (Fig. 2). This observation was made at a single point, which had to be between the first premolar and the second molar, i.e., in the specific area of interest, as mentioned above.

-Statistical Analysis

The distribution of demographic characteristics, anatomical variants and pathologic findings were investigated. Data were described using frequencies (n) and percentages (%) for categorical variables and mean values and standard deviation (SD) for continuous ones. Association of anatomical variants and pathologic findings with patient’s age and gender was assessed by using Chi-square test (X2), or alternatively (when the assumptions for X2 did not hold), Fisher’s exact test, independent samples t-test and one-way analysis of variance (ANOVA). Differences in prevalence between anatomical variants and pathologic findings, based on right or left sinus allocation, were investigated using z-test for comparing two proportions and independent samples t-test. Two-tailed *p-value*s are reported. A *p-value* less than 0.05 (*P*<0.05) indicates a statistically significant result. All analysis was performed using SPSSv.28 (ΙΒΜ Corporation, 2019 Armonk, NewYork, USA).

## Results

Out of the 660 CBCT scans, 268 (40.6%) were male and 392 (59.4%) female patients (age range: 18-92 years, mean age: 64 years).

We first calculated the distribution of findings - based on their location - on either left or right side within the maxillary sinus. No statistically significant difference was found (*P*> 0.05). ( Table 1). Thus, the findings can be further analyzed - as a whole -within each variable, regardless of their location (right or left sinus).

The overall distribution of left and/or right sided anatomical variants and pathological findings in the study sample (660 cases) is clearly shown in  Table 2. AS were found in 255 patients (38.6%), while those might be involved in sinus floor elevation (meeting the previously described criteria) were found in 210 patients (100%). Furthermore, PSAA was detected in 90.2% of patients. Of these, 71.8% and 28.2% were < 1 and ≥ 1 mm in diameter respectively. The mean distance of the PSAA from the sinus floor was 6,4mm (SD: 3.5mm). Hypoplasia was found in 3% of the total sample, sinusitis in 15.6%, odontogenic cysts in 2.7%, retention cysts in 16.1%, and antroliths in 8.6%, while other more uncommon lesions were found in 1.7% (neoplasms and benign fibro-osseous lesions).

The correlation between anatomical variants and pathological findings with patients’ gender is shown in  Table 3. A statistically significant relationship was found between gender and PSAA diameter, PSAA distance from the sinus floor, hypoplasia, sinusitis, and retention cysts (*P*<0.05). In more detail, male patients were more likely to have PSSA ≥1mm in diameter, hypoplasia, sinusitis, and retention cysts. Also, a statistically significant difference was found with the mean PSAA distance from the sinus floor and gender (*P*<0.001). Male patients had on average; a longer distance compared to female patients. No other significant differences were found with the remaining findings and gender (*P*> 0.05).

In the same vein, the correlation between anatomical variants and pathological findings with patients’ age is shown in  Table 4. A statistically significant relationship was found between age and PSAA diameter and sinusitis (*P*<0.05). Specifically, patients aged over 50 years were more likely to have a PSAA diameter ≥1mm, compared to younger patients. Also, sinusitis was more frequent in patients over 70 years of age. No other significant differences were found with the remaining findings and age (*P*> 0.05).

## Discussion

Among all the anatomical variants and pathological findings encountered in the maxillary sinuses, parameters with clinical importance in the surgical procedures of this anatomical area were selected for our study. These included AS and their involvement in a potential sinus floor elevation, as well as the visibility and diameter of the PSAA, and its distance from the sinus floor. The presence of hypoplasia, sinusitis, odontogenic cysts, retention cysts, antroliths, and other less common lesions such as benign fibro-osseous lesions and neoplasms were also evaluated. This was achieved using CBCT, as it has proven to be an ideal imaging tool for identifying subtle anatomical structures such as those of the maxillary sinuses, while providing significant diagnostic information (1).

Regarding AS, their presence is an important anatomical parameter that should be addressed in case of sinus floor elevation. Notably, AS arising from the floor and the inferior half of the lateral walls of the maxillary sinus hinder surgical maneuvers and increase the chances of perforation of the Schneiderian membrane (2-5).

AS greater than 2.5mm in height were included in our study. These were found on either the left or right side in 24.2% and 26.5% of patients, respectively. As for their involvement in possible sinus floor elevation, these were evaluated in 76.9% and 78.4% of the sinus respectively ( Table 1). As shown in  Table 2, 38.6% of patients had AS (either right or left). Of these AS - at least one for each patient - is involved in potential sinus floor elevation (100% of 38.6% of patients). It’s worth noting that the incidence of AS varies widely in the literature and that our results are consistent with those of Abesi *et al*. (2022), Hungerbuhler *et al*. (2019), Kaya *et al*. (2018), and Lana *et al*. (2011) (9-12). Furthermore, no correlation was found between AS and gender or age. This is in line with the results of Bornstein *et al*. (2016) and Hungerbuhler *et al*. (2019) (1,11).

Regarding PSAA, this was detected in 81.1% and 78.8% of either the right or left sinus, respectively, ( Table 1) and in 90.2% of the sample. ( Table 2). It should be mentioned that the visibility of PSAA in a CBCT examination depends on various factors, such as, the diameter of the vessel in relation to the resolution of the imaging system used and, consequently, the voxel size. Hence, to visualize an anatomical structure, it should have dimensions equal to or larger than the voxel size of the system. In particular, the voxel size used in our study was either 0.15 mm or 0.3 mm, depending on each patient’s imaging protocol. Another factor in degerming PSAA is its anatomical position. PSAA to be recognizable must be intraosseous, alternatively, its recognition in CBCT is not feasible. To our knowledge, the visibility of PSAA on CBCT varies widely, ranging from 48.6% to 91.6% (13). Our results align with those of Apostolakis and Bissoon (2013), Rathod *et al*. (2022), Tehranchi *et al*. (2017), and Velasco-Torres *et al*. (2016) (14-17). Also, in our study, the presence of PSAA was found to be independent of the patient’s gender and age. A lack of correlation for these parameters was also found by Ilgüy *et al*. (2013), and Tehranchi *et al*. (2017) (7,16).

Intraoperatively, the presence of the PSAA represents a significant anatomical limitation to the creation of the bony window during sinus floor elevation surgery. Depending on its diameter, injury of this vessel can lead to significant intraoperative bleeding, resulting in restricted visibility, prolonged surgery time, and an increased risk of Schneiderian membrane perforation (6,7). Transection of vessels with a diameter less than 1 mm can cause minimal bleeding and is easily controllable intraoperatively without further complications (10,17,18-21). It has been estimated that for vessels with diameters between 1 - 2 mm, the risk of bleeding during sinus floor elevation is approximately 57%, while for diameters greater than 2 mm, the probability of bleeding increases significantly (6,7). At any rate, maintaining the integrity of vessels in the area is considered essential for proper blood supply, graft incorporation, and subsequent osseointegration of implants (4,22-24).

In our study, PSAA with diameter <1mm - either right or left - was found in 76.7% and 77.9% respectively. Correspondingly, 23.3% and 22.1% had a PSAA diameter ≥1 mm (Table 1). The incidence of PSAA diameter in the total sample (660 patients) was 71.8% for those with diameter <1mm, and 28.2 % for those with diameter ≥1 mm (Table 2). Our results are consistent with those of Velasco-Torres *et al*. (2016) and Ilgüy *et al*. (2013) (7,17). In the study by Apostolakis and Bissoon (2013) - who shared the same unit and exposure settings - the mean vessel diameter was found to be 1.1 mm, with 90% of cases not exceeding 1.5 mm14. Accordingly, Kaya *et al*. (2018) found that the diameter of the PSAA was less than 1 mm in 36.4% of cases (mean 1.04 ± 0.27 mm)10.

Further, we found that PSAA diameter was related to gender and age. More in detail, men were more likely to have PSAA diameter <1 and/or ≥1 mm, as were patients older than 50 years (Tables 3,4). Our results agree with those of similar studies (16,21), and with those of Velasco-Torres *et al*. (2016) who found that vessel diameter increases with age (17). However, other studies have not found a correlation between PSAA diameter and gender or age, so safe conclusions cannot be drawn.

We also measured the mean vertical distance from the inferior bony wall of the PSAA to the sinus floor. This was found to be 6.3 mm and 6.6 mm in either the right or left sinus, respectively ( Table 1) and 6.4 mm of the total sample ( Table 2). This was also found to be significantly greater in males than females; however, it was not related to age. It is worth noting that our measurement was made at a random point in each sinus, where PSAA was most distinct, and should be between the first premolar and second molar, for obvious reasons (sinus floor elevation). It is noteworthy that there is limited research in the literature on this parameter. Apostolakis and Bissoon (2013), after measuring the corresponding distance at five different points for each sinus, arrived at a mean value of 6.4 mm (for each posterior tooth), identical to ours (14). They also found that their findings were not related to gender and age (14). As for the difference in gender bias with our study, this may be attributed to the small sample size used in their study.

Regarding hypoplasia of the maxillary sinuses, this often coexists with a narrower ethmoidal infundibulum and the absence of a normal maxillary ostium (25). It also results in an outward expansion of the lateral walls of the nose, complicating surgical maneuvers (26). In our study, the incidence of hypoplasia was found to be 2.6% in the right and 1.7% in the left maxillary sinus ( Table 1) and in 3% of the sample ( Table 2). Also, this was higher in males than in females, while age was not associated with their rates of occurrence. Our results are consistent with those of Ata-Ali *et al*. (2017) who reported that maxillary sinus hypoplasia ranged from 0.2% to 4.8% (27). However, Amine *et al*. (2020) reported a significantly higher frequency of maxillary sinus hypoplasia (11%) in a Moroccan population, particularly in females (28). Such differences may be attributed to ethnic variations among studies. Of note, hypoplasia has been associated with an increased incidence of maxillary sinusitis (25).

Further on sinusitis, the definition of which - using imaging criteria - varies widely, with reported frequencies ranging from 7.5% to 50% (27). We considered as sinusitis any thickening of the sinus mucosal lining exceeding 5mm, with fluid-gas levels and partial or complete opacification of the sinus. In our study, the incidence of sinusitis was found to be 9.6% in the right and 9.9% in the left maxillary sinus ( Table 1) and in 15.6% of the sample ( Table 2). Similar findings were reported by Pazera *et al*. (2011) and Brullmann *et al*. (2012) (29,30), while Cha *et al*. (2007) found a slightly lower incidence (31). The significantly high incidence (50%) reported by Smith *et al*. (2010) may be attributed to their criteria, as they defined sinusitis, any thickening of the sinus mucosa (32). In addition, we found that the incidence of sinusitis was statistically higher in males than in females, ( Table 3) and in patients older than 70 years compared to younger patients ( Table 4). Regarding gender, our findings are consistent with those of Smith *et al*. (2010), Rege *et al*. (2012) and Vallo *et al*. (2010) (32-34). However, these studies did not found correlation among sinusitis and age. Thus, no definitive conclusions can be drawn about the effect of age.

Regarding odontogenic cysts, these were found in 1.4% and 1.7% of patients in either the right or left sinus, respectively ( Table 1), as well as in 2.7% of the sample ( Table 2). This finding is not significantly correlated to gender and age ( Table 3,  Table 4). Rege *et al*. (2012) also found similar results (33).

Sinus retention cysts are a relatively common pathological finding, with reported incidence ranging from 3.5% to 16.4%. In our study, their incidence was 9% for both sinuses ( Table 1), as well as 16.1% for the total sample ( Table 2), being in agreement with those of Phothikhun *et al*. (2012) (35). Other similar studies reported slightly lower percentages (33,34). Additionally, the frequency of this finding was higher in males compared to females, but without any statistical significance.

On either the right or left side, antroliths were found in 4.2% and 5.3% of the sinuses respectively, and in 8.6% of the total sample studied. Moreover, no statistically significant differences were found with gender and age. Studies in Korean and Taiwanese populations reported antroliths’ presence in 0.99% and 13.8% of their cases, respectively (36). Our results fall within intermediate ranges observed in other studies, such as those by Lana *et al*. (2011) and Rege *et al*. (2012) (12,33). The reported differences in frequencies in the literature may be attributed to racial or climatic factors, since antroliths are more likely to occur in an inflamed mucosa (12,33,36).

Finally, we identified various less common lesions affecting the sinus, such as benign and malignant neoplasms as well as benign fibro-osseous lesions. Their incidence was 0.9% in both the right and left sinus constituting 1.7% of the findings of the total sample studied ( Table 1, Table 2). No correlation with gender or age was found. Similarly, low percentages were reported by Rege *et al*. (2012) (33).

A major limitation of the current study is that the dental status of the patients was not considered. Future studies incorporating this parameter are needed in order to evaluate the correlation between the presence or absence of maxillary teeth and the various anatomical variants and pathological findings of maxillary sinuses.

## Conclusions

CBCT is a valuable imaging technique for assessing the maxillary sinuses for anatomical variants and pathological findings. It also provides precise information about the location of subtle anatomical structures such as AS and PSAA, whose location plays an important role in sinus floor elevation planning. These anatomical structures also showed correlations with gender and age.

## Figures and Tables

**Table 1:** Comparison of results of the anatomical variants and pathological findings based on their location, in the right or left sinus.

Anatomical variations and pathological findings	Location		p-value
	Right	Left	
Antral septa- [n, (%)]	160 (24.2)	175 (26.5)	0.342^1^
Involvement of antral septa in sinus floor elevation- [n, (%)]	123 (76.9)	138 (78.4)	0.298^1^
PSAA- [n, (%)]	536 (81.1)	520 (78.8)	0.215^1^
PSAA < 1mm- [n, (%)]	411 (76,7)	405 (77,9)	0.430^1^
PSAA ≥ 1mm- [n, (%)]	125 (23.3)	115 (22.1)	0.478^1^
PSAA distance from the sinus floor- [Mean, (SD) in mm]	6.3 (3.4)	6.6 (4.9)	0.247^2^
Hypoplasia- [n, (%)]	17 (2.6)	11 (1.7)	0.250^1^
Sinusitis- [n, (%)]	63 (9.6)	65 (9.9)	0.849^1^
Odontogenic cysts- [n, (%)]	9 (1.4)	11 (1.7)	0.653^1^
Retention cysts - [n, (%)]	59 (9.0)	59 (9.0)	0.999^1^
Antroliths- [n, (%)]	28 (4.2)	35 (5.3)	0.368^1^
Other (neoplasms, benign fibro-osseous lesions)- [n, (%)]	6 (0.9)	6 (0.9)	0.999^1^

SD: Standard Deviation 1:z-test for comparing two proportions. 2: independent samples t-test

**Table 2:** Distribution of anatomical variants and pathological findings - regardless of the location of the finding - in the total sample size (N=660).

Anatomical variations and pathologic findings	Descriptive statistical measure
Antral septa- [n, (%)]	255 (38.6)
Involvement of antral septa in sinus floor elevation - [n, (%)]	210 (100.0)
PSAA- [n, (%)]	595 (90.2)
PSAA < 1mm- [n, (%)]	427 (71.8)
PSAA ≥ 1mm- [n, (%)]	168 (28.2)
PSAA distance from the sinus floor - [Mean, (SD) in mm]	6.4 (3.5)
Hypoplasia- [n, (%)]	20 (3.0)
Sinusitis- [n, (%)]	103 (15.6)
Odontogenic cysts- [n, (%)]	18 (2.7)
Retention cysts- [n, (%)]	106 (16.1)
Antroliths- [n, (%)]	57 (8.6)
Other (neoplasms, benign fibro-osseous entities) [n, (%)]	11 (1.7)

SD: Standard Deviation

**Table 3:** Correlation between patients’ anatomical variants and pathological findings and their gender in the total sample (N=660).

Anatomical variations and pathologic findings	Gender Ν (%)		Total	p-value
	Male N= 268	Female N= 392		
Antral septa- [n, (%)]	103 (38.4)	152 (38.8)	255 (38.6)	0.929^1^
Involvement of antral septa in sinus floor elevation - [n, (%)]	87 (100)	123 (100)	210 (100)	-
PSAA- [n, (%)]	239 (89.2)	356 (90.8)	595 (90.2)	0.488^1^
PSAA < 1mm- [n, (%)]	147 (61.5)	280 (78.7)	427 (71.8)	<0.001^1^*
PSAA ≥ 1mm- [n, (%)]	92 (38.5)	76 (21.3)	168 (28.2)	
PSAA distance from the sinus floor- [Mean, (SD) in mm]	7.2 (3.1)	5.8 (3.6)	6.4 (3.5)	<0.001^2^*
Hypoplasia- [n, (%)]	13 (4.9)	7 (1.8)	20 (3.0)	0.024^2^*
Sinusitis- [n, (%)]	58 (21.6)	45 (11.5)	103 (15.6)	<0.001^1^*
Odontogenic cysts- [n, (%)]	6 (2.2)	12 (3.1)	18 (2.7)	0.524^2^
Retention cysts- [n, (%)]	53 (19.8)	53 (13.5)	106 (16.1)	0.026^1^*
Antroliths- [n, (%)]	24 (9.0)	33 (8.4)	57 (8.6)	0.809^1^
Other (neoplasms, benign fibro-osseous lesions)- [n, (%)]	7 (2.6)	4 (1.0)	11 (1.7)	0.132^3^

SD: Standard Deviation 1: Chi square test (Χ2) 2: Independent samples t-test 3: Fisher’s exact test *: Statistically significant, P<0.05 (α=5%)

**Table 4:** Correlation between patients’ anatomical variants and pathological findings and their age in the total sample (N=660).

Anatomical variations and pathologic findings	Age Ν (%)				Total	p-value
	18-30 years N= 74	31-50 years N= 161	51-70 years Ν= 347	71+ years Ν= 78		
Antral septa- [n, (%)]	29 (39.2)	68 (42.2)	128 (36.9)	30 (38.5)	255 (38.6)	0.720^1^
Involvement of antral septa in sinus floor elevation - [n, (%)]	27 (100)	57 (100)	104 (100)	22 (100)	210	-
PSAA- [n, (%)]	63 (85.1)	139 (86.3)	321 (92.5)	72 (92.3)	595 (90.2)	0.062^1^
PSAA < 1mm - [n, (%)]	56 (88.9)	104 (74.8)	219 (68.2)	48 (66.7)	427 (71.8)	0.002^1^*
PSAA ≥ 1mm - [n, (%)]	7 (11.1)	35 (25.2)	102 (31.8)	24 (33.3)	168 (28.2)	
PSAA distance from the sinus floor- [Mean, (SD) in mm]	6.3 (2.9)	6.6 (2.6)	6.4 (4.0)	5.9 (3.1)	6.4 (3.5)	0.680^2^
Hypoplasia- [n, (%)]	0 (0)	3 (1.9)	14 (4.0)	3 (3.8)	20 (3.0)	0.209^2^
Sinusitis- [n, (%)]	5 (6.8)	19 (11.8)	57 (16.4)	22 (28.2)	103 (15.6)	<0.001^1^*
Odontogenic cysts- [n, (%)]	1 (1.4)	3 (1.9)	12 (3.5)	2 (2.6)	18 (2.7)	0.721^2^
Retention cysts- [n, (%)]	13 (17.6)	29 (18.0)	58 (16.7)	6 (7.7)	106 (16.1)	0.192^1^
Antroliths- [n, (%)]	5 (6.8)	13 (8.1)	32 (9.2)	7 (9.0)	57 (8.6)	0.906^1^
Other (neoplasms, benign fibro-osseous lesions)- [n, (%)]	2 (2.7)	2 (1.2)	5 (1.4)	2 (2.6)	11 (1.7)	0.592^3^

SD: Standard Deviation 1: Chi square test (Χ2) 2: One-way analysis of variance (ANOVA) 3:Fisher’s exact test *: Statistically significant, P<0.05 (α=5%)

## Data Availability

The datasets used and/or analyzed during the current study are available from the corresponding author.
